# Vulvar squamous cell carcinoma associated with *Equus caballus* papillomavirus type 2 infection in a Japanese mare

**DOI:** 10.1016/j.tvr.2021.200226

**Published:** 2021-09-17

**Authors:** Nanako Yamashita-Kawanishi, Soma Ito, James K. Chambers, Kazuyuki Uchida, Masato Sato, Hui Wen Chang, Cameron Knight, Frank van der Meer, Takeshi Haga

**Affiliations:** a Division of Infection Control and Disease Prevention, Graduate School of Agricultural and Life Sciences, The University of Tokyo, 1-1-1 Yayoi, Bunkyo-ku, Tokyo, 113-8657, Japan; b Laboratory of Veterinary Pathology, Graduate School of Agricultural and Life Sciences, The University of Tokyo, 1-1-1 Yayoi, Bunkyo-ku, Tokyo, 113-8657, Japan; c Mitsuishi Animal Medical Center, Hokkaido South Agricultural Mutual Aid Association, Hokkaido, Japan; d Graduate Institute of Molecular and Comparative Pathobiology, School of Veterinary Medicine, National Taiwan University, Taipei 106, Taiwan; e Faculty of Veterinary Medicine, University of Calgary, Calgary, Alberta, Canada

**Keywords:** EcPV2, Genital, Vulva, Papillomavirus, Carcinoma, SCC

## Abstract

*Equus caballus* papillomavirus type 2 (EcPV2) infection has been associated with genital squamous cell carcinoma (SCC) development in horses. However, very few reports on EcPV2-associated disease in Asia exist. Our study characterizes pathological and virological features of an EcPV2-associated vulvar SCC from a Japanese mare. Conventional PCR, *in situ* hybridization, reverse-transcriptase PCR and immunohistochemistry confirmed the presence and distribution of EcPV2 within the lesion and suggested that p53 degradation may not be the mechanism by which this virus induces neoplastic transformation. The complete viral sequence in this Japanese case shows near perfect sequence homology with European reference strains of EcPV2, which may be useful when considering the target for future EcPV2 vaccine development. This report also serves to highlight the importance of EcPV2 in female (vulvar) neoplasia, which is less commonly recognized than EcPV2-induced male (penile or preputial) neoplasia. Finally, the SCC described in this mare was an unusual acantholytic variant that has not been reported previously in horses. It is the first report of EcPV2 identified from genital SCC in Asia and underscores the likely worldwide distribution of this virus and its consistent association with equine genital neoplasia.

## Highlights

• This is the first report of *Equus caballus* papillomavirus 2 (EcPV2)-associated genital squamous cell carcinoma (SCC) in Asia.
• This report features EcPV2 in vulvar neoplasia, which is less commonly recognized than EcPV2-induced penile neoplasia.
• Histopathologically, the SCC described in this mare was an unusual acantholytic variant that has not been reported previously.

## 1 Introduction

Papillomaviruses (PVs) are ubiquitous double-stranded DNA viruses with strong tissue and host specificity [1]. To date, nine types of equine PVs (*Equus caballus* PVs or EcPVs) have been characterized from tumors and body fluid samples of horses [[2], [3], [4], [5], [6]]. These EcPV types are classified into the genera Zetapapillomavirus (EcPV1), Dyoiotapapillomavirus (EcPV2, EcPV4, EcPV5) and Dyorhopapillomavirus (EcPV3, EcPV6, EcPV7). Equine PVs 8 and 9 remain unclassified. Equine PVs have been associated with development of cutaneous and mucosal neoplasms in horses. EcPV1 (formerly EqPV) causes self-limiting cutaneous papillomas, typically of the muzzle and distal limbs [2,7,8], while EcPV2 has been associated with development of benign and malignant equine genital neoplasms such as papillomas, carcinomas *in situ*, and squamous cell carcinomas (SCCs) [6,[9], [10], [11], [12]]. EcPVs 3, 5, and 6 have been associated with aural plaques [3], EcPV7 has been identified in penile masses from a gelding [3] and EcPV8 has been detected in cases of generalized papillomatosis [5], and in viral papillomas, plaques and SCCs of the inguinal region [13]. EcPV9 has been detected in semen collected from a thoroughbred stallion with a penile lesion [4].

Among these EcPV types, EcPV2 is the most studied because of the frequently reported association between infection and genital neoplasia [6,[9], [10], [11]]. However, studies related to EcPV2 have been conducted mainly in Europe, North America, and Australasia [6,[9], [10], [11],[14], [15], [16]]. In Japan, only one study describing EcPV2 infection exists to date; it describes EcPV2 sequence detection in an equine guttural pouch SCC lesion [17]. In this current report we describe pathological and virological characteristics of an EcPV2-associated vulvar SCC in a Japanese mare, and add to the knowledge of this virus's pathogenesis and worldwide distribution in horses.

## 2 Materials and methods

### 2.1 Case description, sample collection and haematoxylin and eosin staining

**Fig. 1:**
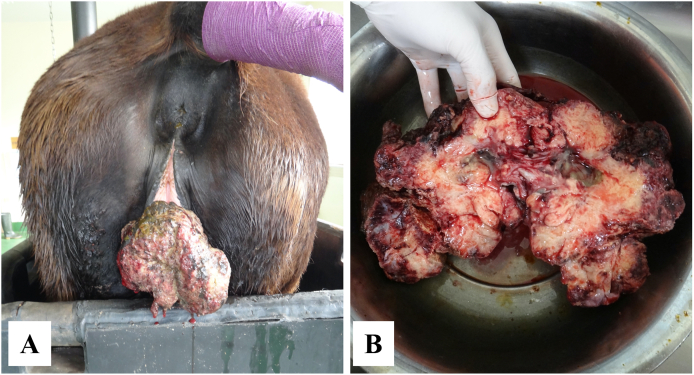
Vulvar mass in a 28 year old Thoroughbred mare. (A) Mass *in situ* prior to surgical excision. (B) Mass after excision and bisection.

A rapidly growing ventral vulvar mass developed in a 28-year-old Thoroughbred mare kept in Hokkaido Prefecture, Japan (Fig. 1A). The mare had traveled within Japan but had no history of international travel, and she had foaled a total of 14 times between 1998 and 2016. The mass was approximately 20 cm in diameter and had a multifocally ulcerated superficial surface. After surgical excision under sedation and caudal epidural anaesthesia the mass was bisected longitudinally, revealing a tan, fleshy core with scattered foci of presumed necrosis (Fig. 1B). No recurrence was seen after tumor excision. The mare died three months post-surgery, however the cause of death was not recorded.

After bisection, one half of the excised mass was stored at −30 °C for later DNA extraction and the other was fixed in 10% buffered formalin. The fixed tissue was embedded in paraffin, sectioned, and stained with haematoxylin and eosin for histologic examination [18].

### 2.2 DNA extraction and PCR

For PV DNA detection, genomic DNA was extracted from the frozen tissue sample using the QIAamp DNA Mini Kit (QIAGEN, Hilden, Germany) following the manufacturer's protocol. The success of DNA extraction was validated by conventional PCR using a primer pair targeting equine beta-actin named Equine β-actin (F/R) [19]. Forty nanograms of extracted DNA were used for PCR reaction. PCR was performed using DNA polymerase, KOD FX Neo (Toyobo, Shiga, Japan) following the manufacturer's instructions. For electrophoresis of the PCR-amplified products a 2% agarose gel with GeRed (﻿Biotium, Inc., Fremont, CA, U.S.A.) was used. The bands and 100 bp ladder marker (WATSON Bio Lab, San Diego, CA, U.S.A) were visualized by UV-exposure (Optima Inc., Tokyo, Japan). For PV detection, three primer pairs were used: BPV1/2 L1 subA modified (F/R) to detect BPV1/2 [19], 498 EcPV1 L1 (F/R) for EcPV1, and 445 EcPV2 L1 (F/R) for EcPV2 (Supplementary Table 1). Distilled water was used as a negative control. Synthesized DNA including the primer sequences BPV1/2 L1 subA modified (F/R) and 498 EcPV1 L1 (F/R) was used as a positive control for this primer pair, to diminish sample contamination. For the 445 EcPV2 L1 (F/R) primer pair, DNA extracted from a confirmed EcPV2-positive equine penile papilloma was used. PCR was performed by three-step cycling condition as follows: pre-denaturation at 94 °C for 2 min, denaturation at 98 °C for 10 s, annealing at respective temperatures for 30 s, followed by extension at 68 °C for 30 s. Annealing temperatures for each respective primer pair can be found in Supplementary Table 1.

### 2.3 Whole-genome sequencing of EcPV2 and phylogenetic analysis

To obtain the complete sequence of EcPV2 identified in this mare's mass, three primer pairs dividing the full EcPV2 genome into three amplicons were used for PCR. Primer pairs were named EcPV2 415 (F)/EcPV2 3115 (R), EcPV2 2714 (F)/EcPV2 5705 (R) and EcPV2 5264 (F)/EcPV2 543 (R) (Supplementary Table 1). An 1.2% agarose gel was used for electrophoresis of the amplicons and they were purified using the NucleoSpin Gel and PCR Clean-up kit (Macherey-Nagel, Düren, Germany). Gel-purified PCR products were subjected to both direct sequencing and sub-cloning. Direct sequencing was carried out ﻿with BigDye™ Terminator v3.1 Cycle Sequencing Kit (Thermo Fisher Scientific, Waltham, MA, U.S.A.) and ABI 3130xl Genetic Analyzer (Applied Biosystems, Foster City, CA, U.S.A.). Sequencing was performed bidirectionally as previously described [19] by applying sequencing primers designed in this study (Supplementary Table 1). Gel-purified samples were also cloned into the pCR-XL-2 TOPO cloning vector (Invitrogen, Carlsbad, CA, U.S.A.). Sequence data were analyzed using Molecular Evolutionary Genetics Analysis Version X software [20] and respective open reading frames (ORFs) were determined using the ORF Finder tool from the National Center for Biotechnology Information (NCBI) (https://www.ncbi.nlm.nih.gov/orffinder/). Sequences were compared with the reference EcPV2 sequence (GenBank accession number: EU503122) using the Needle program [21] of Emboss Pairwise Alignment Algorithms (http://www.ebi.ac.uk/emboss/align/). A phylogenetic tree was constructed based on EcPV2 E6 nucleotide sequence of 44 isolates (74–400 nt of the reference EcPV2 sequence, 327bp) available in the GenBank database including the Japanese isolate identified in this study. The tree was built with ﻿Molecular Evolutionary Genetics Analysis Version X software (MEGA X) [20] by the neighbor-joining method [22] with bootstrap replicates of 1000. ﻿The evolutionary distances were computed using the maximum composite likelihood method [22] and are in units of the number of base substitutions per site.

### 2.4 *In situ* hybridization (ISH)

Localization of EcPV2 genes in tumor tissue was assessed by *in situ* hybridization (ISH). Digoxigenin (DIG)-labeled EcPV2 E6, E7, and L1 genes were obtained by PCR amplification of genomic DNA using the PCR DIG Probe Synthesis Kit (Roche Diagnostics, Mannheim, Germany) according to the manufacturer's protocol. After enzyme digestion with Carezyme III: Pronase Kit (Biocare Medical, Concord, CA, U.S.A.) at room temperature and autoclave pre-treatment in citrate buffer, tissue sections were hybridized with one of the DIG-labeled DNA probes.

### 2.5 Immunohistochemistry (IHC)

Immunohistochemistry (IHC) was carried out using three primary antibodies targeting PV L1 antigen (clone BPV1/1H8+CAMVIR; Abcam, Cambridge, MA, U.S.A), retinoblastoma protein (pRb) (clone G3-245; BD Pharmingen, Tokyo, Japan), and p53 (clone FL-393; Santa Cruz Biotechnology, Santa Cruz, CA, U.S.A.). The numbers of immunopositive tumor cells were counted in 5 high-power fields (x400).

### 2.6 Reverse-transcriptase PCR (RT-PCR)

To investigate viral involvement in tumor development, mRNA expression of early and late PV genes was identified by reverse-transcriptase PCR (RT-PCR). Total RNA from the frozen tumor sample was extracted using the RNeasy mini kit (Qiagen), following the manufacturer's instructions. Genomic DNA was eliminated using recombinant DNase I (Takara, Kusatsu, Japan) and reverse transcription was performed using the PrimeScript RT Reagent Kit (Takara) according to the manufacturer's protocol. Detection of three early oncogenes (E2, E6, E7) and a late gene (L1) was verified by using four pairs of primers listed in Supplementary Table 1. For the L1 detection, a primer pair named 445 EcPV2 L1 (F/R) was used (Supplementary Table 1). Synthesized cDNA, DNase-treated RNA, and genomic DNA from this mare's mass were used as PCR reaction templates for each primer pair. DNase-treated RNA was included to ensure the success of DNA elimination in the cDNA template. PCR reactions were carried out using KOD FX Neo DNA polymerase (Toyobo), annealing temperatures can be found in Supplementary Table 1. RT-PCR results were confirmed by gel electrophoresis and sequencing, as described above.

## 3 Results

### 3.1 Histopathological diagnosis

Histological examination of the vulvar mass revealed submucosal invasion by discrete to coalescing lobules of neoplastic epithelial cells separated by a markedly desmoplastic fibrous stroma (Fig. 3A). Neoplastic lobules were composed of a rim of basaloid epithelial cells with disorderly central keratinization and, frequently, suprabasilar acantholysis giving rise to pseudoluminal structures (Fig. 3B). Mitotic figures were common. A diagnosis of squamous cell carcinoma (SCC) was made.

### 3.2 EcPV2 detection and sequence characteristics

**Fig. 2:**
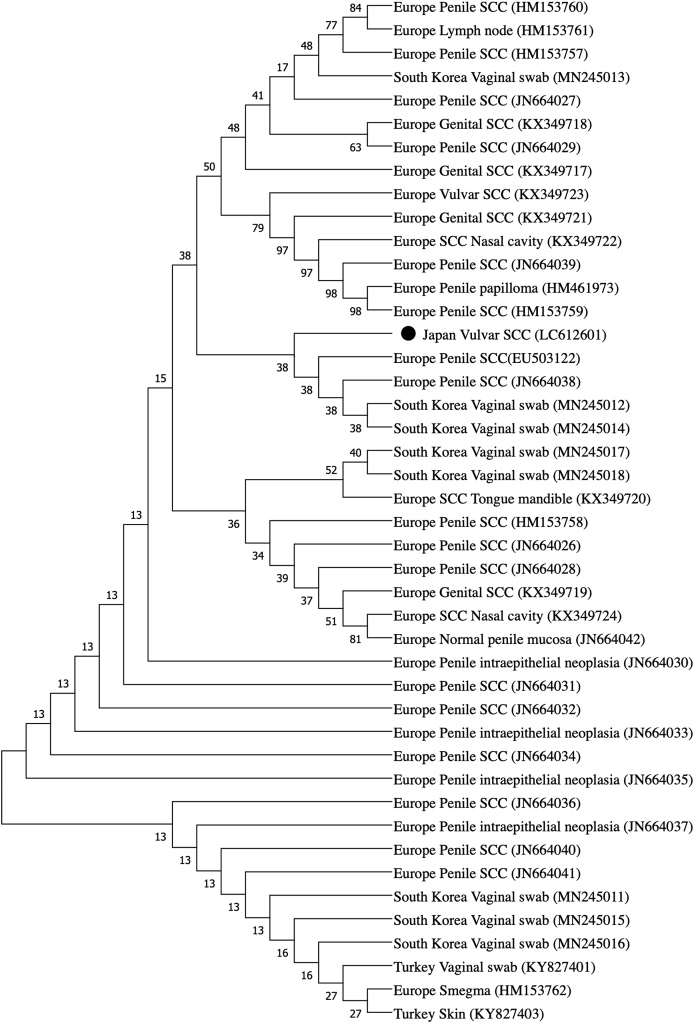
Phylogenetic tree of EcPV2 E6 ﻿ A phylogenetic tree including 44 EcPV2 isolates was constructed by the neighbor-joining method based on E6 nucleotide sequence (74–400 nt of the reference EcPV2 sequence, 327bp). The percentage of replicate trees in which the associated taxa clustered together in the bootstrap test (1000 replicates) is shown next to the branches. Origins of geographical area and tissue/lesion are noted. Respective GenBank accession numbers are shown in parentheses. The EcPV2 E6 sequences identified in the present study are noted by closed circle. ﻿Abbreviations: SCC, squamous cell carcinoma.

EcPV2 L1 could be detected in the tumor tissue using PCR. Neither BPV1/2 L1 nor EcPV1 L1 were detected by PCR (data not shown). Direct sequencing revealed that the EcPV2 identified in this mare's tissue samples shared high sequence homology with the EcPV2 reference sequence EU503122. The full genome of EcPV2 identified in this mare's mass was 7083bp in length and 99.94% (7798/7803 nt) identical to the EcPV2 reference sequence. The nucleotide sequence homology based on each ORF was E1: 99.95% (1883/1884 nt); E2: 100% (1251/1251 nt); E6: 100% (618/618 nt); E7 100% (354/354 nt); L1: 99.93% (1499/1500 nt); L2: 99.81% (1557/1560 nt); long control region (LCR): 100% (634/634 nt). A total of three amino acid substitutions were observed in the E1 (N51D), L1 (R489K), and L2 (D299A). Sequence results of the respective clones were 100% identical to those of the direct sequencing results. When compared with the two European EcPV2 sequences (GenBank accession numbers: EU503122 and HM461973), our case had nucleotide substitution in the E2 binding site (E2BS) (ACC-N6-GGT) [23] of the L2 (nucleotide position: 4838, T/C). The complete EcPV2 sequence of this mare's sample was deposited in the GenBank/EMBL/DDBJ database under accession number LC612601. The phylogenetic tree of the E6 nucleotide sequence showed that the Japanese isolate branched near the European isolates (Fig. 2).

**Fig. 3:**
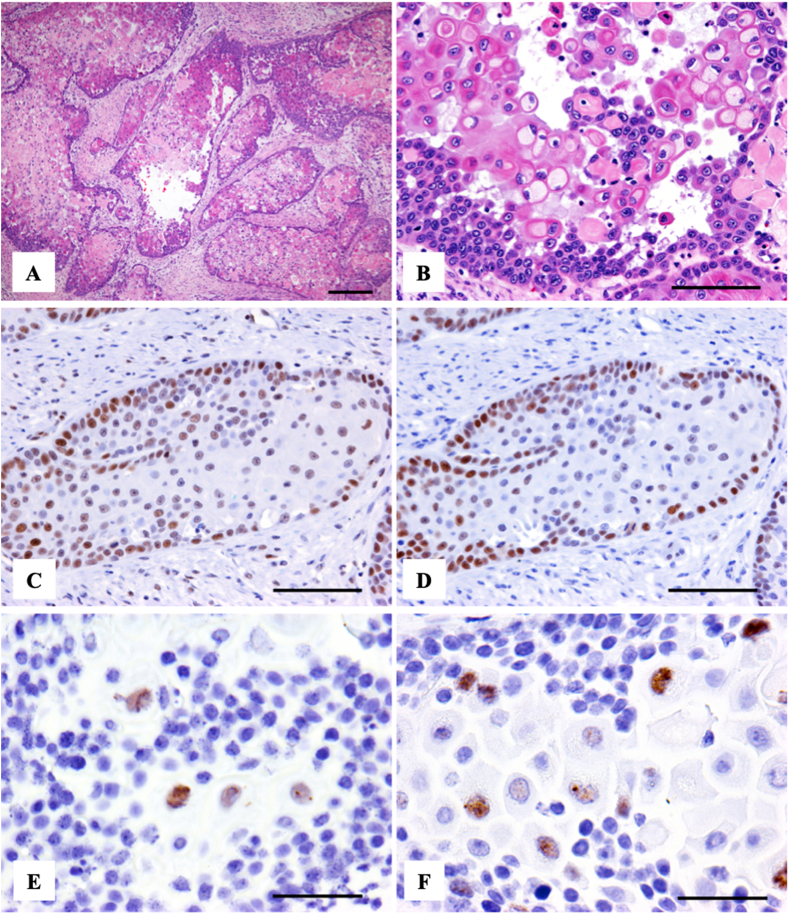
Photomicrographs of *Equus caballus* papillomavirus type 2 (EcPV2)-associated vulvar squamous cell carcinoma from a 28 year old mare. (A) Lobules of neoplastic squamous cells infiltrate the submucosa. Haematoxylin and eosin (HE) staining. Bar, 100 μm. (B) Tumor cells show disorderly keratinization and suprabasilar acantholysis. HE staining. Bar, 100 μm. (C) The nuclei of approximately 25% of tumor cells are immunopositive for pRb. Immunohistochemistry. Bar, 100 μm. (D) The nuclei of approximately 20% of tumor cells are immunopositive for p53. Immunoreactivity is predominantly located within the basal layer. Immunohistochemistry. Bar, 100 μm. (E) Strong and punctate nuclear hybridization signals for E6 in a subset tumor cells. *In situ* hybridization Bar, 50 μm. (F) Strong and punctate nuclear hybridization signals for L1 in a subset tumor cells. *In situ* hybridization Bar, 50 μm.

### 3.3 Positive signals for EcPV2 genes by *in situ* hybridization

Using ISH, signals for EcPV2 E6, E7, and L1 genes were detected in a subset of tumor cells (Fig. 3E and F). Positive signals were mostly located in cells in the center of tumor foci and less frequently in cells of the basal layer. Signals for L1 were more numerous and stronger than those for E6 and E7. Signals for all three probes, where present, were predominantly strong and filled the nucleus (“diffuse nuclear signal”), although weaker, punctate signals were occasionally observed.

### 3.4 Detection of tumor suppressor proteins by IHC, but not of PV L1 antigen

Tumor cells were negative for PV L1 antigen; 26% of tumor cells were immunopositive for pRb (Fig. 3C); and 20% of tumor cells were immunopositive for p53 (Fig. 3D). The p53-positive tumor cells were predominantly located in the basal layer of tumor islands, while the distribution of pRb-positive tumor cells was more widespread.

### 3.5 mRNA expression of EcPV2 genes

**Fig. 4:**
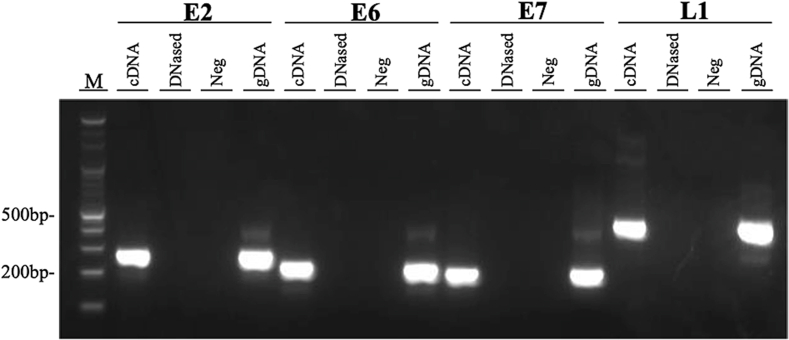
EcPV2 mRNA expression by reverse-transcriptase PCR (RT-PCR). Gel electrophoresis results confirmed cDNA (mRNA) expression of EcPV2 E2, E6, E7, and L1 by RT-PCR in this mare's tissue sample. A single band around the expected size of 282 bp (E2), 234 bp (E6), 214 bp (E7), and 445 bp (L1) was confirmed by RT-PCR. Abbreviations: bp, base pair; cDNA, complementary DNA; DNased, DNase-treated RNA; gDNA, genomic DNA; M, 100bp marker; Neg, negative control.

Gel electrophoresis of PCR products showed no residual contamination of viral genomic DNA in the cDNA samples, confirming the viral mRNA expression of L1, E2, E6, and E7 in this mare's sample (Fig. 4).

## 4 Discussion

Although the presence of EcPV2-associated genital SCC in Asia is not unexpected, it is worthwhile for Asian veterinarians to know that this virus is now confirmed to be present. Similarly, it is important that veterinarians globally consider EcPV2 infection in their differential diagnosis for equine genital tumors, regardless of whether or not EcPV2 has been reported in their geographic region.

SCC is the most common neoplasm of the equine penis and prepuce [[24], [25], [26]]. Numerous studies have now focused on the role of EcPV2 in the development of equine penile/preputial SCCs [9,11,15,27,28]. However, there are still relatively few reports describing EcPV-2 vulvar-associated SCC in mares [6,16,24,28,29]. Since EcPV2 is possibly sexually transmitted, female genital SCC is a logical counterpart to penile and preputial SCC. It is important, therefore, to accumulate and report data on the incidence of EcPV2-associated female genital neoplasia and, again, for veterinarians to include EcPV2 in their differential diagnosis.

The vulvar SCC in this mare was histologically characterized by acantholysis of tumor cells, which is different from previously reported cases of EcPV-associated SCC in horses [9,10,30,31]. An acantholytic variant of SCC, in which partially keratinized tumor cells detach from the centres of neoplastic lobules to form pseudoglandular or pseudoluminal structures, has been described in domestic mammals [32], humans, and even a human case of HPV-associated vulvar SCC [33]. The significance of this acantholytic variant in terms of aggressiveness and any association with HPV is debated and remains uncertain, however this current case is the first description of this histologic pattern in any EcPV-2 induced equine lesion.

This is also the first report describing the complete gene sequence for EcPV2 identified in Asia. The mare had mating history including stallions imported from the U.S.A. Currently, the two complete EcPV2 sequences available in GenBank originated in Europe (accession numbers: EU503122 and HM461973) but no sequences from the U.S.A. were available. The nucleotide sequence of the complete EcPV2 genome from this Japanese mare's SCC showed very high similarity to both European references (99.94% for EU503122 and 99.05% (7729/7803 nt) for HM461973). Although there is a limitation in the sequence data numbers in GenBank, E6 phylogenetic tree showed that our Japanese EcPV2 isolate branched near the European isolates (Fig. 2). This sequence similarity and phylogenetic results may be due to the recent introduction of EcPV2 to Japan through importation of European horses, or to genomic stability of EcPV2. Our finding is in contrast to that of a swab-based Korean study that found sequence variation in the PV E6 gene [34].

The majority of PVs have the E2BS located in the LCR, but BPV types 19 and 21 retain this binding motif in the L2 ORF [35], as do both European EcPV2 reference isolates. E2 is known to regulate viral replication and oncogene (E6 and E7) expression [36]. However, any association between rapid lesion growth and the specific nucleotide substitution observed in the E2BS of EcPV2 L2 of this mare's tumor is unknown.

To confirm the presence of EcPV-2 in this mare's mass we used several complementary tests. Conventional PCR was used to demonstrate EcPV2 sequences and to rule out the presence of EcPV1, BPV1 and BPV2, all of which are commonly found in equine skin lesions. A limitation of this study is that we did not screen for less common EcPVs (3 through 9) by using specific primers, or for unknown PVs by using consensus primers. We therefore cannot rule out the possibility that another EcPV might be present in the tumor mass. Except for this mare, no other horses in the same breeding farm developed neoplasia of the external genitalia. As EcPV2 infection has been identified in asymptomatic horses [28,34], further molecular epidemiological studies are needed to identify the prevalence of EcPV2 in horses in Japan.

To determine whether the EcPV2 detected in this mare's mass was involved in tumorigenesis we performed ISH, RT-PCR and IHC. *In situ* hybridization localized EcPV2 genes *E6* and *E7* within tumor sections (Fig. 3E and F), and RT-PCR confirmed mRNA expression of viral E2, E6 and E7 (Fig. 4). Papillomaviral E6 and E7 genes are viral oncogenes that promote host epithelial cell differentiation through disruption of cell cycling. This is enhanced by accidental integration of viral *E2* into the host genome, which disrupts the *E2* and releases the viral promoters of the PV oncogenes E6 and E7 [37,38]. Overexpression of PV E6 and E7 is a consistent feature of PV-associated malignant neoplasms in humans [39] and their efficiency at transforming cells is increased when they are expressed together [40,41]. Taken overall, our ISH and RT-PCR results are consistent with other studies, and support the involvement of EcPV2 infection in tumor development in this mare.

Human papillomaviral *E6* and *E7* oncogene products act by degrading or disrupting host tumor suppressor proteins pRb and p53 [42,43]. Immunohistochemistry may be used to investigate levels of pRb and p53 within lesions and, in humans, a reduction in both suggests that a tumor is PV-induced. A previous IHC-based study of feline PV-associated neoplasia described that pRb was considered to be reduced within a lesion if less than half of the cells contained intense immunostaining, and p53 was considered *not* to be reduced if more than 20% of the cells contained intense immunostaining [44]. Based on these criteria, the SCC from this mare showed reduction of pRb immunostaining but not of p53 immunostaining. This combination is consistent with certain PV-induced SCCs of humans [45], horses [11], and cats [44]. Furthermore, our p53 results are also consistent with an IHC-based study of 39 cases of equine SCC that showed no significant difference in p53 expression between EcPV-2 positive penile SCCs and EcPV2-negative penile SCCs or head and neck SCCs (Knight CG, unpublished data). Unlike carcinogenic HPVs [46], EcPV2 does not retain retinoblastoma binding domain (LXCXE) in the E7 [6]. A previous study showed that PVs lacking LXCXE motif binds and degrades pRb by using the C-terminus of E7 [47]. In cats, a previous study presented that *Felis catus* papillomavirus type 2 (FcaPV2) which harbors LXCXE motif, was not detected from the SCC lesions that contained reduced pRb immunostaining [44], suggesting that the loss of pRb may be no specific for a PV cause. The significance in the reduced pRb immunosignal and the etiological association of EcPV2 needs to be further elucidated. To the best of our knowledge, our case report is also the first to evaluate pRb and p53 by IHC in an equine vulvar SCC.

Using IHC there was no immunostaining of papillomaviral L1 antigen in this mare's tumor tissue. However, the presence of EcPV2 L1 within the tumor was demonstrated by conventional PCR, ISH and RT-PCR (Fig. 4). The absence of L1 antigen immunostaining may be explained in two ways. Firstly, the antibody may not have recognized EcPV2 L1, or it may have been under the detection limit. The monoclonal antibody used in our IHC experiment was raised against BPV1 L1, however, our previous study on EcPV1 succeeded in detecting PV antigen in the equine papilloma lesion using the same antibody [7]. Secondly, integration of PV DNA into the host genome may occur. This integration increases the chance of malignant transformation but, at the same time, reduces viral replication and production of PV capsid proteins; for this reason PV-infected malignant tumors are typically non-productive, and IHC directed at L1 antigen is unsuccessful [48].

## 5 Conclusions

In summary, this study characterizes pathological and virological features of an EcPV2-associated vulvar SCC from a Japanese mare. It is the first report of EcPV2 identified from genital SCC in Asia and, as such, should underscore the likely worldwide distribution of this virus and its association with equine genital neoplasia. This is something that all veterinarians should consider in their differential diagnosis, regardless of whether or not EcPV2 has been reported in their geographic region. This report also serves to highlight the importance of EcPV2 in vulvar neoplasia, which is less commonly recognized than EcPV-2-induced penile or preputial neoplasia. The virus in this Japanese case was fully sequenced and shows a high sequence homology with European reference strains of EcPV2, which may be useful when considering the target for future EcPV2 vaccine development. A variety of molecular testing confirmed the presence and distribution of EcPV2 within the lesion described in this report, excluded the possibility of other common PVs found in horses, and suggested that p53 degradation may not be the mechanism by which this virus induces neoplastic transformation. Finally, the SCC described in this mare was an unusual acantholytic variant that has not been reported previously in horses. This study will expand the knowledge of EcPV2 and its role in equine cancer.
